# Controlling the Optical Properties of Transparent Auxetic Liquid Crystal Elastomers

**DOI:** 10.1021/acs.macromol.3c02226

**Published:** 2024-02-19

**Authors:** Emily J. Cooper, Matthew Reynolds, Thomas Raistrick, Stuart R. Berrow, Ethan I. L. Jull, Victor Reshetnyak, Devesh Mistry, Helen F. Gleeson

**Affiliations:** † School of Physics and Astronomy, University of Leeds, Leeds LS2 9JT, United Kingdom; ‡ Taras Shevchenko National University of Kyiv, Kyiv 03680, Ukraine

## Abstract

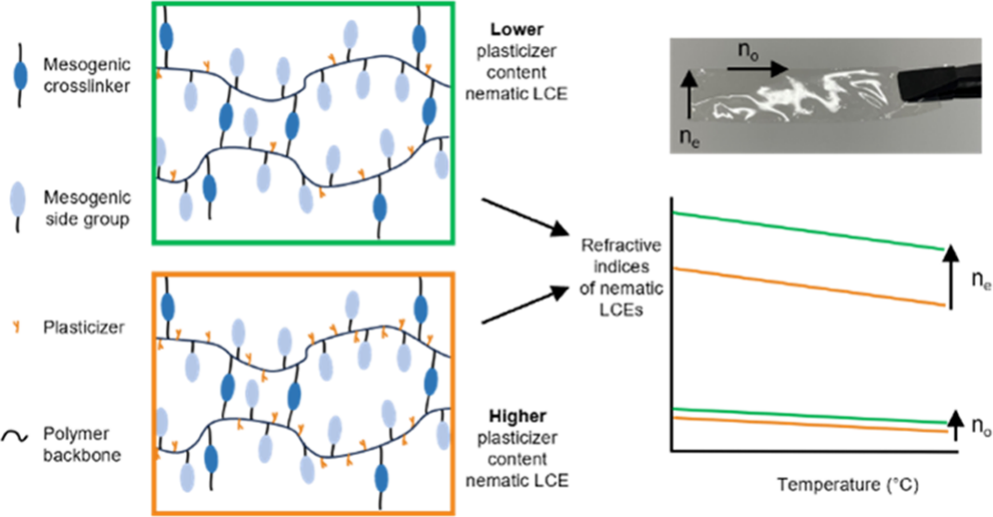

Determining the tunability of the optical coefficients, order parameter, and transition temperatures in optically transparent auxetic liquid crystal elastomers (LCEs) is vital for applications, including impact-resistant glass laminates. Here, we report measurements of the refractive indices, order parameters, and transition temperatures in a family of acrylate-based LCEs in which the mesogenic content varies from ∼50 to ∼85%. Modifications in the precursor mixture allow the order parameter, ⟨*P*_2_⟩, of the LCE to be adjusted from 0.46 to 0.73. The extraordinary refractive index changes most significantly with composition, from ∼1.66 to ∼1.69, in moving from a low to high mesogenic content. We demonstrate that all LCE refractive indices decrease with increasing temperature, with temperature coefficients of ∼10^–4^ K^–1^, comparable to optical plastics. In these LCEs, the average refractive index and the refractive index anisotropy are tunable via both chemical composition and order parameter control; we report design rules for both.

## Introduction

Liquid crystal elastomers (LCEs) are networks formed from cross-linked liquid crystal polymers. Since the first LCE was synthesized by Finkelmann in 1981,^1^ the community has continually developed and demonstrated polymerization routes and formulations which enable simpler production, and greater control and versatility over composition and physical properties.^1−3^ Owing to the coupling of liquid crystalline and elastomeric properties, LCEs demonstrate shape actuations responsive to thermal, optical, and chemical stimuli,^4^ and also unique mechanical phenomena including negative Poisson’s ratio behavior, also referred to as auxeticity,^5^ and soft elasticity.^6^ In fact, the first synthetic material to display molecular auxetic behavior was an acrylate LCE developed by Mistry et al.^20^ This material also has the unique benefit, among auxetic materials, of being transparent, offering the potential for the mechanical advantages of auxetic LCEs, including resistance to impact and delamination, to be applied to optical systems. The most commonly proposed applications for LCEs rely on their actuation and shape change, though an increasing number, including some sensors and medical devices, depend on mechano-optical behavior.^7−9^ For auxetic LCEs, potential applications also include impact-resistant glass. The optical properties of LCEs, particularly the values of the ordinary (*n*_o_) and extraordinary (*n*_e_) refractive indices and the birefringence (Δ*n*), therefore affect the proposed function. Despite their importance, there are rather few reports on the optical properties of LCEs. In this paper, we report the measurement and control of the refractive indices of a family of transparent auxetic LCEs where the average refractive index is modified chemically, while the anisotropy is determined by manipulation of the order parameter of the precursor mixture.

One of the earliest proposals of an optical device using LCEs was a contact lens concept by Amigó-Melchior et al., which used the optical anisotropy of a monodomain LCE to produce a bifocal lens suitable for treating conditions such as astigmatism and presbyopia.^10^ The refractive indices of LCEs are also critical to their sensitivity and appearance in optical strain sensing and mechanochromic devices which are based on the photoelastic (strain-induced birefringence) and selective reflection (strain-dependent chiral nematic pitch) effects.^9,11−13^ More broadly, where an LCE is integrated into an optical or display device, for instance, for haptic,^14^ cleaning,^15^ or protection purposes,^16^ the material refractive indices and transparency will affect the optical quality of the whole device. The refractive indices of optical plastics are widely reported in the range of 10–40 °C to understand and optimize suitability for applications at near-ambient conditions.^17^ Clearly, for optical applications of LCEs, knowledge of the material’s optical properties and methods for controlling them is critical; we address both in this work.

Although measurements of refractive indices have been widely reported for low-molar-mass liquid crystals, measurements of the individual refractive indices of LCEs have rarely been reported. Broer et al. used an Abbé refractometer to measure the temperature dependence of densely cross-linked liquid crystal networks and focused on the refractive indices’ temperature dependence in the glassy phase, concluding that the decrease in refractive indices with temperature was driven by the changing density of the network.^18,19^ Their studies demonstrated that the final networks’ refractive indices were controlled via the temperature at which polymerization was performed; however, the studies did not consider how refractive indices of the networks could be controlled via formulation. Several papers report the birefringence of LCEs, for instance, as a function of temperature; however, as highlighted above, knowledge of the refractive indices themselves is important.^5,20^ Varanytsia et al. analyzed the reflection spectra of a chiral nematic acrylate LCE designed for lasing, to deduce that *n*_o_ and *n*_e_ took values between 1.50–1.53 and 1.65–1.59, respectively, over a temperature range from ∼23 to 75 °C.^21^ This technique can be extremely accurate for chiral liquid crystals where excellent alignment can be achieved and a fit can be made to the reflection spectra to deduce the optical coefficients.^22^ Optical diffraction has also been reported for measurements of refractive index modulation in azo-doped siloxane LCEs, with modifications of birefringence of the order of 10^–2^.^23^ These few studies indicate the challenge in measuring refractive indices of LCEs and, therefore, the lack of knowledge of how to control refractive indices using methods such as formulation.

**Figure 1:**
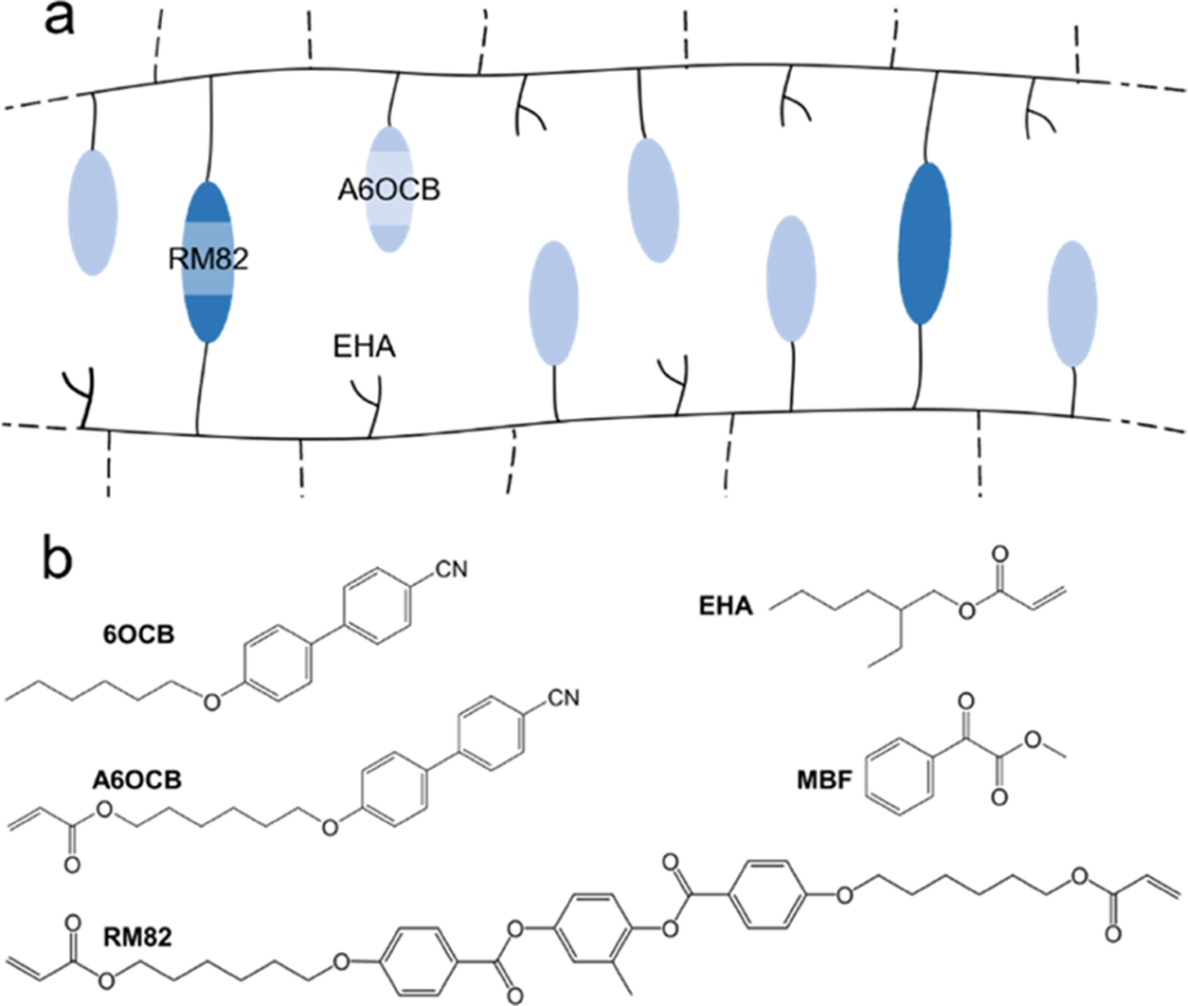
(a) Schematic of the acrylate-based side-chain liquid crystal elastomer (LCE) family used in this work. The polyacrylate backbone is shown as a black, continuous line, the side groups are nonmesogenic 2-ethylhexyl acrylate (EHA) and mesogenic A6OCB, while the cross-linker is the diacrylate reactive mesogen, RM82. (b) Chemical structures of the additional components included in the LCE precursor mixture, with the mesogen, 6OCB, and the photoinitiator methylbenzolformate (MBF) also shown.

In this paper, we report accurate refractive index measurements for a family of acrylate-based LCEs and demonstrate tuning of the optical properties by simple chemical modification of the polymer network. We use the family of acrylate-based nematic LCEs synthesized by Mistry et al., the components of which are shown in Figure 1a,b.^20^ This system of LCEs features mesogenic and nonmesogenic components allowing simple control of refractive indices via formulation. The polymerized LCEs can also be templated with either nematic or isotropic symmetries and using a choice of polymerization conditions, which allows further control of optical properties. The isotropic variants of these materials have been shown to mechanically behave like conventional isotropic elastomers but with exceptionally large photoelastic constants, meaning they have excellent potential as optical strain sensors.^9^ By contrast, the nematic materials from this family of LCEs exhibit negative Poisson’s ratio (or “auxetic”) regimes, representing the first synthetic, transparent materials that are auxetic at a molecular level.^20^ As auxetic materials are resistant to both impact and delamination, this discovery offers optical materials with novel mechanical properties, that are expected to be useful in a variety of new application areas.

Here, the composition of our LCEs has been systematically varied by including different proportions of a nonmesogenic monomer, 2-ethylhexyl acrylate (EHA) (see Figure 1a). We describe how composition modifications influence the nematic to isotropic transition temperature of the precursor mixture and the room-temperature order parameters and refractive indices of the resultant LCE. As the refractive indices will depend on both the chemical composition of the LCE and the nematic order parameter,^24^ we determine the optical properties and the order independently. The order parameter of the LCE can be controlled by both the temperature at which the nematic precursor mixture is polymerized and the proportion of mesogenic material. This can also be understood from Maier–Saupe theory, since the order parameter *S* is a function of , and the parameter *U* depends on the fraction of the mesogenic material. Therefore, *S* is a function of both temperature and mole fraction, *S*(*T*, mole fraction).^25^ Finally, we show that composition and order parameters influence the refractive indices and birefringence of the final elastomer. Hence, we provide some design rules for optical tunability in LCEs.

## Experimental Section

### LCE Film Preparation

The liquid crystal elastomers used in this paper are based on those previously reported.^20,26^ The elastomers comprise a nonmesogenic material, 2-ethylhexyl acrylate (EHA), a mesogenic cross-linker, 1,4-bis-[4-(6-acryloyloxyhexyloxy)benzoyloxy]-2-methylbenzene (RM82), and a monofunctional mesogenic side group, 6-(4-cyano-biphenyl-4′-yloxy)hexyl acrylate (A6OCB). A photoinitiator, methyl benzoylformate (MBF), is also added to initiate polymerization of the network. An additional nonreactive mesogenic material, 4-cyano-4′-hexoxybiphenyl (6OCB), is included in the precursor mixture to extend the nematic phase prior to polymerization, allowing high-quality, monodomain films to be produced; the 6OCB is washed from the final LCE.^5,20^ The molecular structures of the components of the LCEs are shown in Figure 1b. The RM82, A6OCB, and 6OCB were supplied by Synthon Chemicals GmbH (Bitterfeld-Wolfen, Germany), and the EHA and MBF were supplied by Sigma-Aldrich (Gillingham, U.K.).

The preparation of the LCE films has been described in detail previously.^5^ Briefly, for each material prepared, the mesogenic materials are first mixed for 5 min at 120 °C. Once cooled to approximately 40 °C, MBF and EHA are added via a pipet, and the mixture is stirred for a further 2 min. The mixture is then filled into prepared molds at 40 °C, using the capillary effect. These molds are assembled using a 100 μm thick strip of Melinex as a spacer between a glass substrate and a 250 μm Melinex substrate, both coated with poly(vinyl alcohol) and rubbed. For nematic LCEs, the filled mold then rests in the nematic phase at room temperature for 20 min to ensure good alignment prior to being irradiated with a 2.5 mW/cm^2^ UV light source for 2 h, as described previously.^25^ For isotropic LCEs, the material was instead held at an elevated temperature in the isotropic phase and irradiated with UV light for 2 h. The LCE is then removed from the mold and washed overnight in a methanol and dichloromethane mixture to remove the 6OCB. Following the overnight wash, the elastomer is then left at ambient conditions to dry by evaporation of the washing solvents. Previous work on this family of acylate LCEs has shown no porosity, investigated using AFM down to ∼5 nm.^20^

In this work, the composition of the nematic LCEs was altered through the quantity of EHA added to the precursor mixture, thereby varying the mole percent (mol %) of the total mesogenic materials, RM82 and A6OCB, within the final network. Specifically, the mol % ratio of RM82:A6OCB in the elastomer remained constant (1:7), and the mol % of the total mesogenic content for the nematic LCEs was varied between 51 and 84%. The lower limit was dictated by phase separation in the nematic liquid crystal elastomer (nLCE), while the upper limit was a consequence of the glass transition temperature approaching room temperature. We use a nomenclature that describes the nematic LCEs by their mol % of mesogenic content, so a nematic LCE with 62 mol % mesogenic content is referred to as nLCE-62. For reference, nLCE-62 was the starting material previously studied^26^ within this acrylate LCE family.

### Thermal Behavior

The phase transitions of both the precursor mixtures and the final LCEs were studied by using Differential Scanning Calorimetry (DSC). Samples of between 5 and 9 mg were contained in aluminum pans, loaded into a TA Instruments Q20 with an RCS90 cooling system, and measured for three cycles with a heating/cooling rate of 10°C/min. The nematic to isotropic transition temperature, *T*_NI_, of the unpolymerized precursor mixtures was measured as the onset of the transition peak on cooling with run cycles between 100 and −60 °C. The glass transition temperature, *T*_g_, of the LCEs (no 6OCB) was determined from runs between 250 and −40 °C, measured by the inflection on cooling. For all samples, the average transition temperatures across the three cycles of cooling were used, with the uncertainty deduced from the standard deviation.

### Order Parameters

Measurements of the nematic order parameter were made using polarized Raman spectroscopy following a methodology that has been described in detail elsewhere.^27,28^ Briefly, a Renishaw inVia Raman microscope with a 532 nm solid-state laser of 2.5 mW power was used to determine the Raman spectra of the elastomer samples in a backscatter geometry. A 20× objective was used for all elastomers, except the phase-separated nLCEs, where a 50× objective was required to ensure that measurements were performed only on regions with nematic order. The phase separation of nematic samples with a low mesogenic content is discussed later.

The elastomer samples were placed onto a glass slide and fixed on the rotational stage of the Raman microscope, allowing measurements to be made at 10° intervals over a full 360° rotation of the sample. Intensity data were recorded with the analyzer both parallel and perpendicular to the input light polarization, allowing a depolarization ratio to be calculated. The ∼1606 cm^–1^ peak, corresponding to the breathing mode of the phenyl group, was selected for analysis as it has previously been shown to satisfy all of the requirements for the determination of the order parameters of liquid crystals.^27,28^

Equations 1 and 2 describe the scattering intensities for parallel and perpendicular polarizations, respectively, as a function of sample rotation, θ, differential polarizability ratio, *r*, and the uniaxial order parameters, ⟨*P*_2_⟩ and ⟨*P*_4_⟩.^27,28^
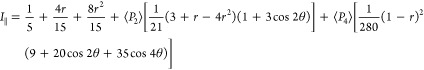
1
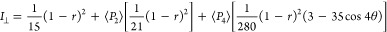
2

Fitting the measured depolarization ratio, , as a function of the sample rotation extracts the polarizability ratio together with the order parameters, ⟨*P*_2_⟩ and ⟨*P*_4_⟩ to an accuracy of 0.05.^27^ These order parameters are investigated experimentally for this uniaxial system to determine the role of the LCE composition on order. An example of the fitting is shown in Figure S1, using nLCE-56.^28^

### Refractive Indices

Undoubtedly, the most accurate methodology used to determine the refractive indices of liquid crystals is via an Abbé refractometer, a technique that has been widely used for low-molar-mass materials at temperatures below ∼80 °C. Refractometry techniques are also often used for measuring the refractive index of transparent plastics.^17^ This approach was used by Broer et al. to study densely cross-linked liquid crystal networks but has not so far been used for LCEs.^18,19^ The fact that the method requires a large (∼1.5 cm × 3.5 cm), uniform, highly transparent area means that it can only be used with relatively large, well-aligned, monodomain samples, as are obtained for our LCEs with the fabrication techniques described above. The acrylate-based LCEs considered in this work have an ∼94% transparency at 589 nm, measured using transmission spectroscopy, shown in Figure S2. The transparency of our LCEs therefore enabled measurements of temperature-dependent refractive indices using a 60/ED Abbé Refractometer by Bellingham and Stanley and a Neslab RTE-4 refrigerated circulating bath.

The Abbé Refractometer operates by determining the critical angle for total internal reflection of the sample with respect to a reference prism and is often used for studying the refractive indices of transparent liquids and solids.^29^ The LCE films were placed on the prisms of the Abbé Refractometer, with care taken to ensure good contact, and the illumination was provided by a sodium lamp of wavelength 589 nm. The refractive indices, *n*_o_ and *n*_e_ were measured at temperature intervals of roughly 2 °C between 55 to 25 °C. Multiple measurements were taken at each temperature, to calculate an average of each refractive index value with an error calculated from the standard deviation.

Reflection Spectroscopy was employed to determine the refractive index of poly(2-ethylhexyl acrylate), referred to here as pEHA. An Oceanview Spectrometer connected to an Olympus microscope enabled reflection spectroscopy of a thin (∼9 μm) film of pEHA contained between sealed glass slides. The methodology has been described in detail elsewhere;^30^ the glass separation was first measured with an accuracy of 0.1 μm and the gap was then filled with pEHA. The material’s refractive index was determined with an accuracy of ∼2% by fitting to the reflection spectrum.^30^ The film was held at 25.4 °C using a Linkam LTS 350 hot stage connected to a Linkam TMS 93 temperature controller; this temperature was chosen to allow comparison to the average refractive index data of nematic LCEs from the Abbé Refractometer.

## Results and Discussion

### Transition Temperatures

**Figure 2:**
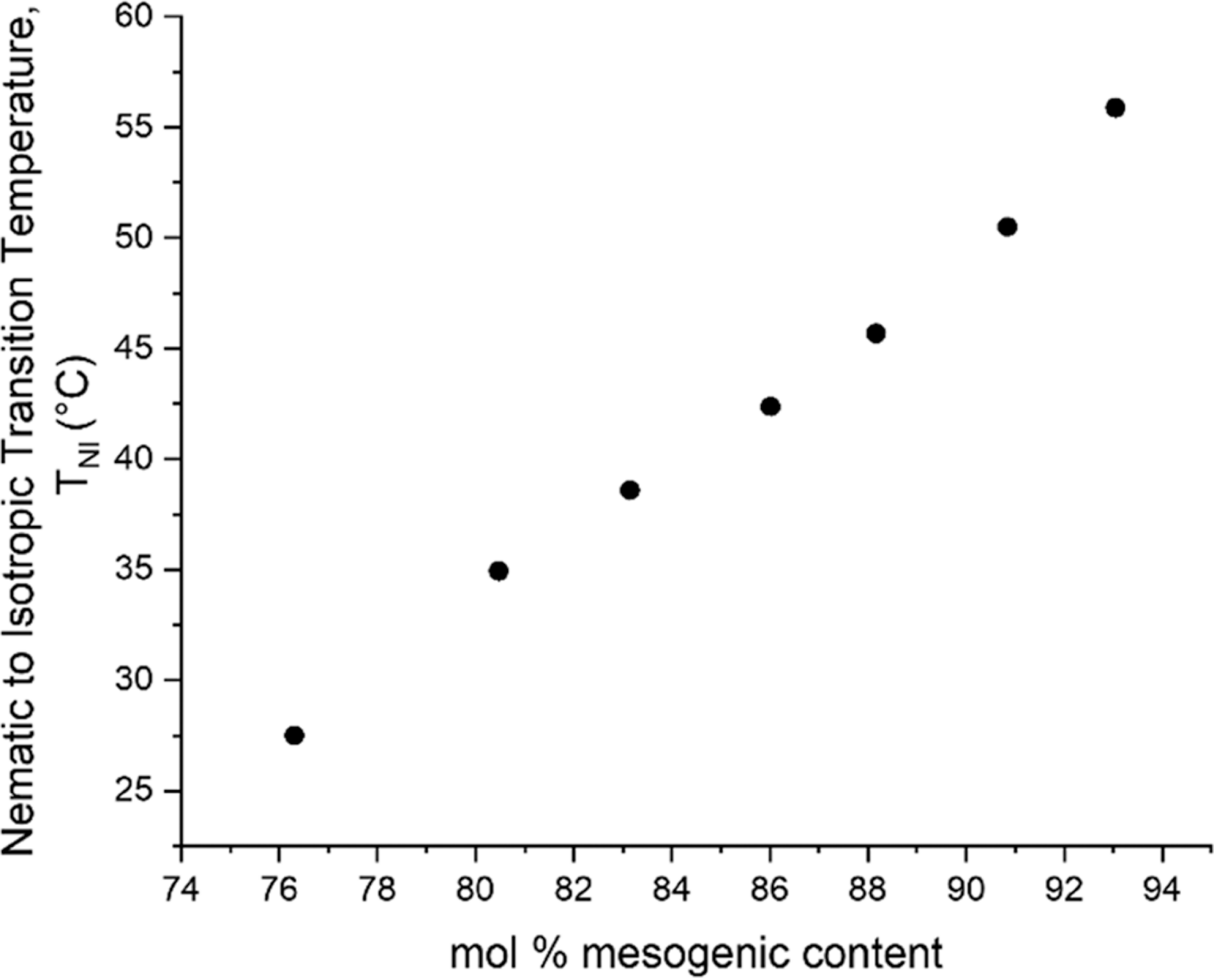
Dependence of the nematic to isotropic phase transition temperature (*T*_NI_) of the LCE precursor mixtures on their mesogenic content.

Thermal transitions are critically important to the processability, properties, and applications of an LCE. With this system of materials, the difference between the polymerization temperature and the nematic to isotropic transition temperature affects the degree of order, which is imprinted onto the final LCE. Additionally, the glass transition temperature of the final LCE impacts the temperature window over which it can be used for a given application. Figure 2 shows *T*_NI_ for the LCE precursor mixture over the range of the mesogenic content studied. As would be expected, the precursors with greater mesogenic content (which includes the nonreactive 6OCB) have higher values of *T*_NI_. As all of the nematic LCEs were polymerized in the nematic phase at room temperature, this therefore means that for LCEs with higher mesogenic content, their network was formed deeper into the nematic phase.

The linear dependence of *T*_NI_ on the mol % of mesogenic content can be understood from Maier Saupe theory as follows.^25^ It is known that , where *U*(*T*_NI_) describes the magnitude of the anisotropic part of the intermolecular interaction; this is temperature-independent in Maier Saupe theory. It is reasonable to assume that the intermolecular interaction parameter *U*(*T*_NI_) is a linear function of the mole fraction of the mesogenic content. Then, from , the nematic to isotropic transition temperature, *T*_NI_, should also be a linear function of the mole fraction of mesogenic content, which is supported by Figure 2.

**Figure 3:**
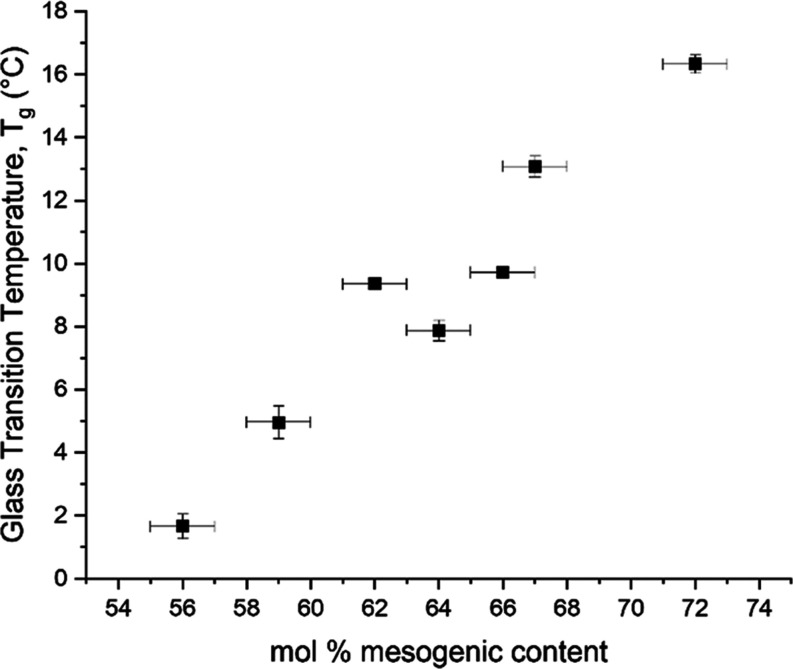
Dependence of the glass transition temperature (*T*_g_) of polymerized LCEs on their mesogenic content. Each material has a *T*_g_ lower than room temperature.

Similarly, the *T*_g_ of the polymerized LCEs also increased with mesogenic content (see Figure 3). Note, the values for mesogenic content shown in Figures 2 and 3 are different since the precursor materials also include the unreactive 6OCB component which is washed out of the final LCE. The trend of increased *T*_g_ with an increased mesogenic content in Figure 3 can be understood in two ways. First, the materials with lower mesogenic content contain a greater fraction of EHA. As pEHA has a *T*_g_ of ca. −69 °C,^31^ increased amounts of EHA can be expected to lower the glass transition temperature of the copolymer blend. Second, in this system, an increase in quantities of EHA also decreases the mol % of the cross-linking group RM82, thus increasing the mobility of the network and lowering the material’s *T*_g_. All of the LCEs studied here exhibit a *T*_g_ below room temperature (∼22 °C in our laboratories), so they are soft rubbers at room temperature. Care must be taken when loading soft materials into the Abbé Refractometer, ensuring good contact with no air bubbles trapped between the sample and the prism. As previously seen for this family of acrylate LCEs,^5,9^ no *T*_NI_ was observed via DSC for any of the networks, here measured up to 250 °C. Above this temperature, at temperatures nearing 330 °C, these materials have previously been shown to begin degrading, with still no clear evidence of a nematic to isotropic phase transition.^5^

### Temperature-Dependent Refractive Indices

**Figure 4:**
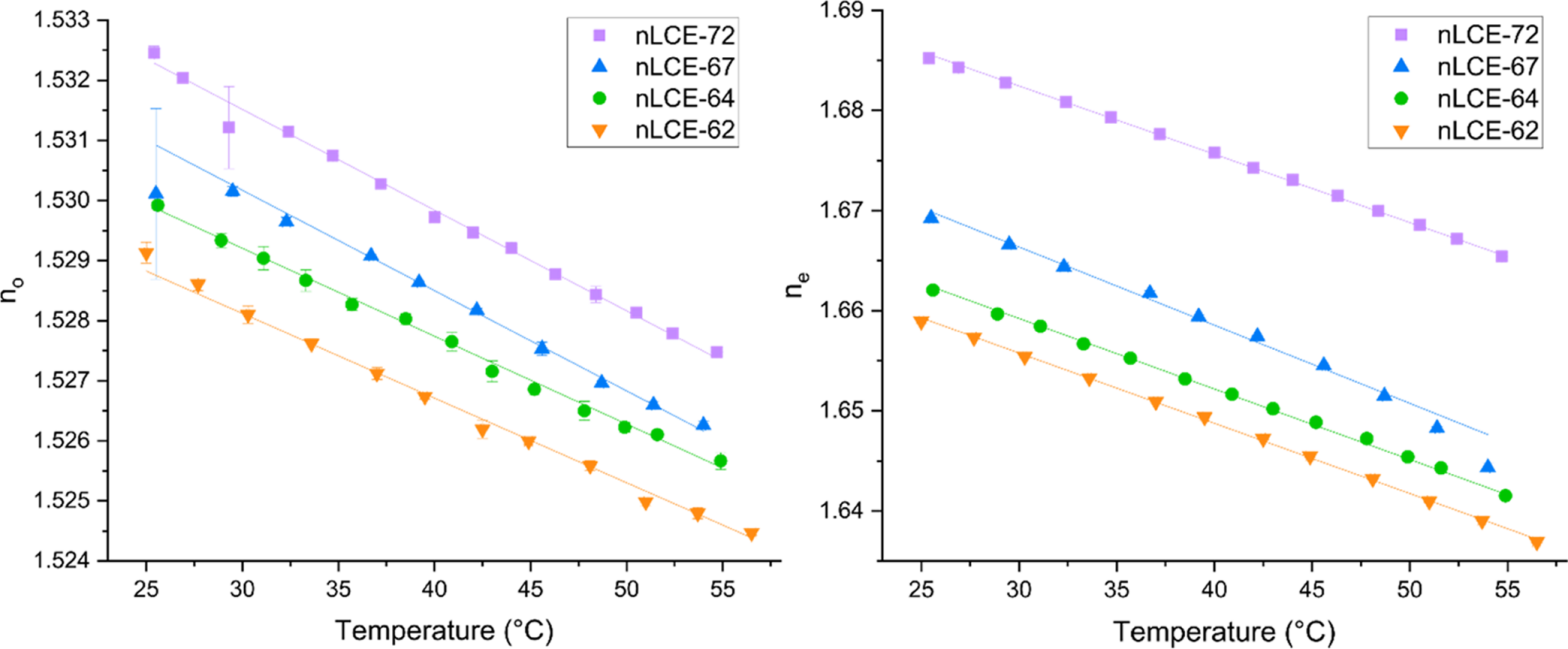
Temperature dependence of the ordinary and extraordinary refractive indices, *n*_o_ and *n*_e_, respectively, for nematic LCEs with 72 mol % (purple squares), 67 mol % (blue triangles), 64 mol % (green circles), and 62 mol % (orange triangles) mesogenic content.

Figure 4 shows the ordinary and extraordinary refractive indices, *n*_o_ and *n*_e_, respectively, determined for each nLCE as a function of temperature. A greater birefringence *(*Δ*n* = *n*_e_ – *n*_o_) is observed for an increased mesogenic content in the LCE, driven by larger changes in *n*_e_ than *n*_o_ (see Figure S3). Indeed, a 10% change in mesogenic content causes *n*_e_ to vary by ∼0.026 while *n*_o_ varies by only ∼0.003.

For each material in Figure 4, the refractive indices, *n*_o_ and *n*_e_, linearly decrease by ∼0.005 and ∼0.015, respectively, over the ∼30 °C window studied. Such a linear decrease in refractive index with temperature is typically attributed to changes in density and is common for many optical plastics. Linear fits can be used to determine a temperature coefficient of refractive index, d*n*/d*T*, for each of the LCEs, as shown in Table 1.^17^ The temperature coefficients of the ordinary d*n*_o_/d*T* and extraordinary d*n*_e_/d*T* refractive indices are found to be between −1 × 10^–4^ and −8 × 10^–4^ K^–1^, which are of the same order as many optical plastics.^17^ Moreover, the temperature coefficient of the average refractive index for the LCEs, d*n*_av_/d*T*, where , is ca. – 3.5 × 10^–4^ K^–1^, comparable to reported experimental values of coefficients for acrylate elastomers, which are between −4.2 × 10^–4^ and −4.5 × 10^–4^ K^–1^.^32,33^

**Table 1:** Temperature Coefficients of Refractive Index for the Ordinary, Extraordinary, and Average Refractive Indices, , , and , Respectively, for the nLCEs Studied

	temperature coefficient of refractive index (×10^–4^ K^–1^)		
nLCE mol % mesogenic content (±1)	d*n*_o_/d*T*	d*n*_e_/d*T*	d*n*_av_/d*T*
72	–1.68 ± 0.01	–6.82 ± 0.05	–3.47 ± 0.02
67	–1.67 ± 0.03	–7.8 ± 0.3	–3.94 ± 0.01
64	–1.46 ± 0.02	–7.05 ± 0.08	–3.37 ± 0.02
62	–1.41 ± 0.03	–7.02 ± 0.05	–3.41 ± 0.02

It is noteworthy that the clearly linear behavior of the refractive indices of the LCEs over the temperature range studied is quite distinct from the usual temperature dependence of refractive indices of liquid crystals.^24^ Specifically, although *n*_e_ reduces with increasing temperature in all liquid crystals, its behavior is not usually linear and *n*_o_ typically increases with increasing temperature. Such behavior is driven by the reduction in order parameter of nematic liquid crystals with increasing temperature. The fact that the nLCEs reported here have exceptionally high *T*_NI_ values explains why the linear fits with negative gradient describe the temperature dependence of all refractive indices so well; the variation driven by the temperature dependence of the order parameter is negligible over the temperature range studied. Similar behavior was reported by Broer et al. in the glassy phase of liquid crystal networks and also attributed to a density change in the network.^18,19^

**Figure 5:**
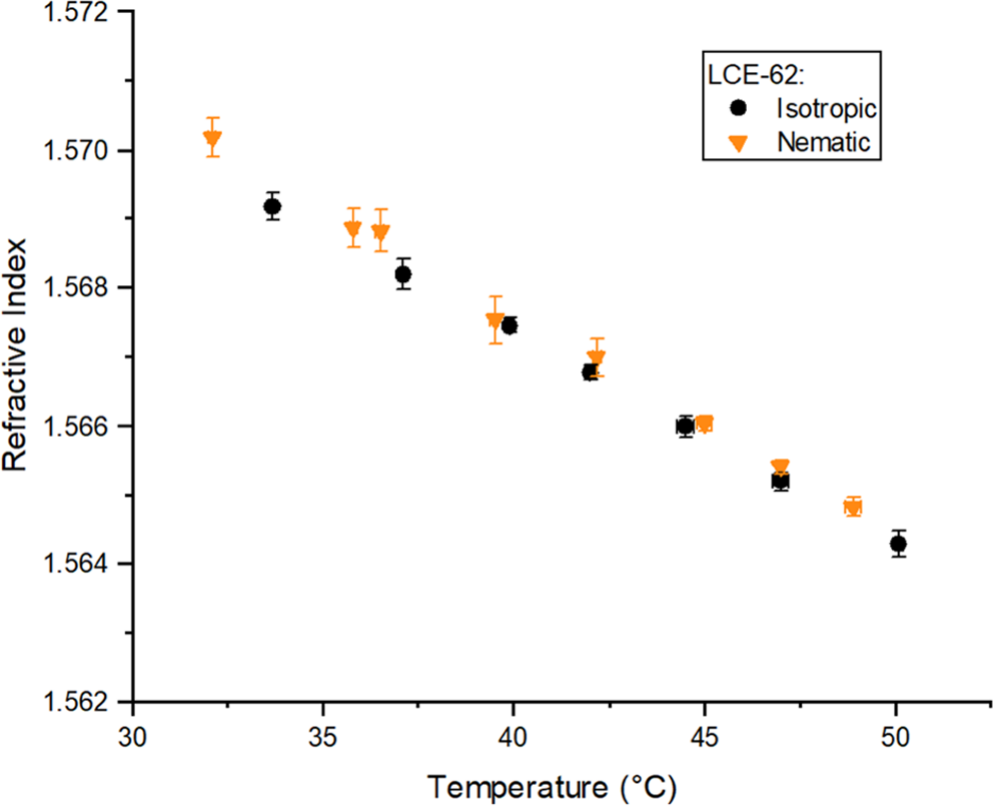
Average refractive index deduced for a nematic LCE (orange triangles) and measured directly for an isotropic LCE (black circles) of the same chemical composition, with 62 mol % mesogenic content. The indices are measured across the same temperature range for samples templated with different order (nematic or isotropic).

Interestingly, in this system of LCEs, it is possible to compare the average refractive index of a nematic LCE calculated from , with the values measured for the isotropic version of the LCE, n_iso_ (chemically identical but polymerized in the isotropic phase).^9^Figure 5 demonstrates the excellent agreement between the n_av_ and *n*_iso_ for LCE-62, validating the use of the geometric average of the anisotropic refractive indices to calculate an average refractive index and showing that this index is decoupled from the effects of the temperature of polymerization and order, i.e., it is purely dependent on chemical composition. This result also confirms that the optical anisotropy should be solely related to the order parameter, a factor examined in the following section. The lack of influence of order parameter on the average refractive index can be further understood by looking at the mesogenic and nonmesogenic contributions to the refractive indices of the material, where the mesogenic contribution will involve temperature-dependent order parameter and density, and nonmesogenic will involve only temperature-dependent density. From considering the dielectric tensors, the average refractive index,  can be derived as independent of the order parameter.^34^ This finally results in an approximately linear temperature dependence of the averaged refractive index, within the relatively small temperature interval 30–50 °C.

### Dependence of Refractive Indices and Order Parameters on Composition

**Figure 6:**
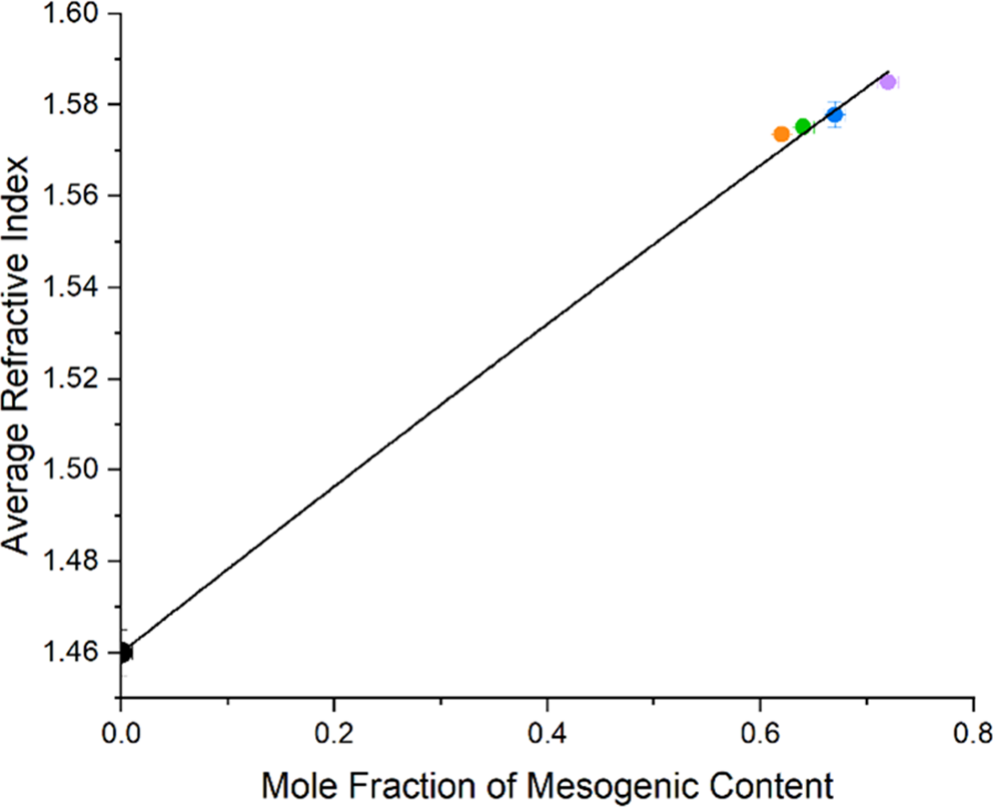
Average refractive index of nLCEs and pEHA measured at 25.4 ± 0.4 °C for various mole fractions of mesogenic content. The straight line fit to the data is using eq 3. The data corresponds to nematic LCEs with 72 mol % (purple circle), 67 mol % (blue circle), 64 mol % (green circle), and 62 mol % (orange circle) mesogenic content.

The role of composition on the refractive indices of the nematic LCEs is investigated by first considering how the average refractive index depends on the relative concentrations of the mesogenic and nonmesogenic components. Figure 6 shows the average refractive index of the LCEs as a function of the mole fraction of the mesogenic content at 25.4 ± 0.4 °C. The refractive index of pEHA (zero mesogenic content) was measured to be 1.46 ± 0.01 at 25.4 ± 0.1 °C using reflection spectroscopy, as described earlier.

Reis et al.^35^ describe how the refractive index of a two-component mixture depends on the concentration of each component. Equation 3, adapted from the Newton equations,^35^ relates the average refractive index of the LCE to the mole fraction of mesogenic content, *M*, the refractive index of pEHA, *n*_pEHA_, and a fitting parameter that describes refractive index of the purely mesogenic material, *n*_mesogen_.
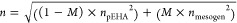
3

Figure 6 demonstrates a good fit to eq 3, with the fitting parameter *n*_mesogen_ = 1.634 ± 0.002 representing the average refractive index of an LCE with 100% mesogenic content. Therefore, the average refractive index of a nematic LCE, and the refractive index of isotropic LCEs, can be tuned through composition and can be predicted using mixing equations together with a knowledge of the refractive indices of the pure mesogenic and nonmesogenic components.

**Figure 7:**
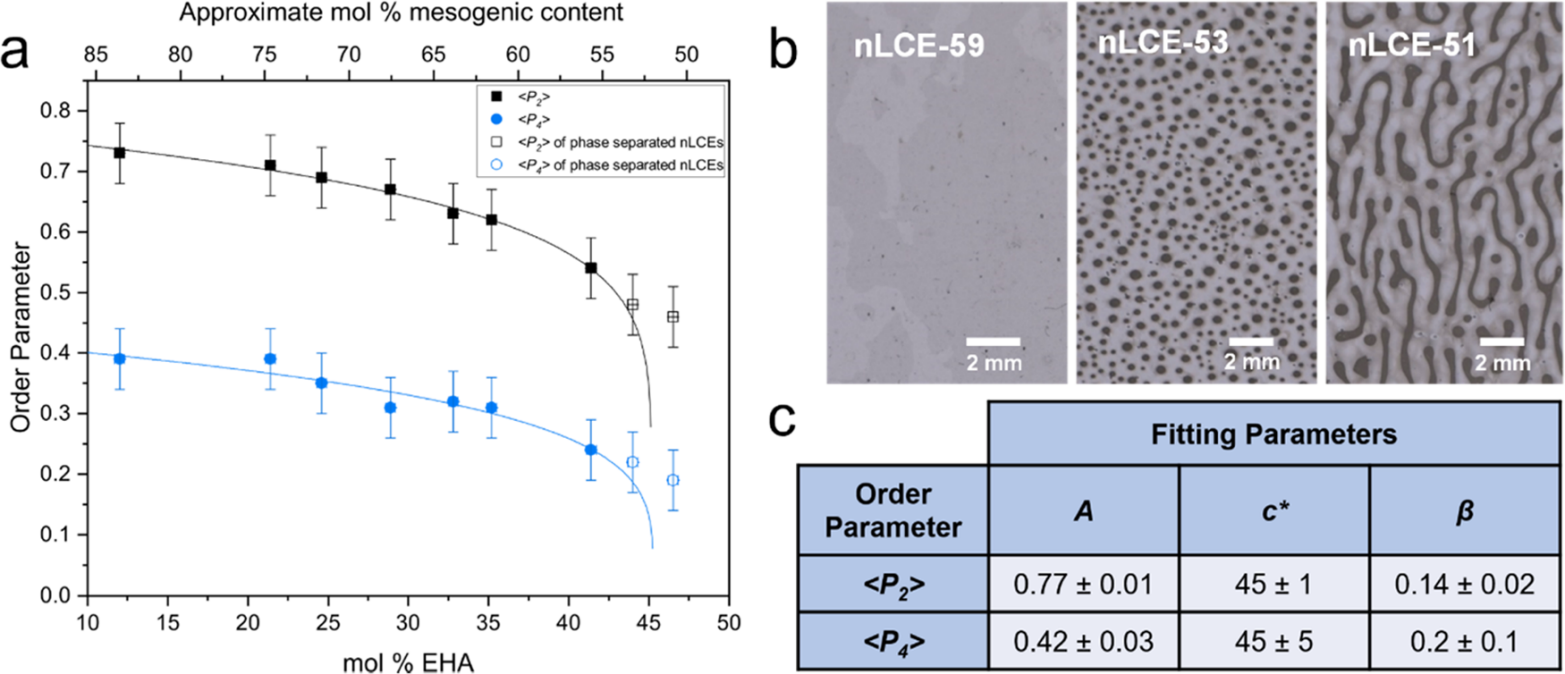
(a) Nematic order parameters of the nLCEs plotted both as a function mol % of nonmesogenic component, EHA, (lower axis) and the approximate mol % of mesogenic content (upper axis). The fit to the data uses eq 5 and is applied to only data where no phase separation was observed (<45% EHA). (b) Images of films of nematic LCEs with compositions below and above the critical concentration of EHA (* in eq 5). Images show films with 59, 53, and 51 mol % mesogenic content from left to right. The left-most image shows a uniform nematic phase with excellent alignment and no phase separation, whereas the two samples on the right display different degrees of phase separation. In the latter two films, the order parameters were deduced by focusing only on the nematic regions. (c) Fitting parameters for ⟨*P*_2_⟩ and ⟨*P*_4_⟩ according to eq 5 (the line fits are shown in (a)).

We now consider whether, in addition to controlling the average refractive index through composition, it is also possible to control the birefringence of the nLCEs. As mentioned previously, materials in this family of LCEs can be templated to have either nematic or isotropic symmetries. For LCEs polymerized in the nematic phase, the magnitudes of the resultant order parameters are expected to be dependent on both the material formulation and the temperature (with respect to *T*_NI_) at which the system was polymerized; the latter is consistent with Broer et al.’s findings for glassy liquid crystal networks.^18^ To explore this, we first consider how the order parameters of the nLCEs vary with composition over a wide range of mesogenic content. Figure 7a shows the order parameters ⟨*P*_2_⟩ and ⟨*P*_4_⟩ determined for each of the nLCEs as functions of their nonmesogenic content. The validity of the measurements is confirmed by considering their agreement with Maier Saupe theory; see Figure S4. In Figure 7a, the order parameters are seen to increase with an increased mesogenic content. This is consistent with the increased difference between the polymerization temperature and the precursor’s *T*_NI_ (higher mesogenic content results in higher *T*_NI_, Figure 2).

The order parameter data can be explained in detail by considering an analogy with the more commonly explored temperature dependence that is described by well-known Onsager-type models, such as the Haller model, as given in eq 4.^24^
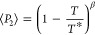
4

In eq 4, *T* is the temperature, T* is a critical temperature, typically just above *T*_NI_, and β is a material-specific fitting constant.^36^ A similar approach can be invoked that describes how the scalar order parameter depends on the concentration, *c*, of a nonmesogenic component,
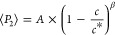
5where c* is a critical concentration above which the system is isotropic, β is again a material-specific fitting constant, and *A* is a parameter to allow freedom of the *y*-axis intercept in the fitting. Such a model has been used by Barbero et al. to describe the dependence of order in liquid crystal systems undergoing a *cis*–*trans* isomerization of azobenzene groups.^37^ In their work, the conformational change of the azobenzene groups changed the concentrations of mesogenic (trans) and nonmesogenic (cis) content. We have adapted their approach and used eq 5 to analyze the dependence of the nLCE order parameters, ⟨*P*_2_⟩ and ⟨*P*_4_⟩, on the concentration of the nonmesogenic groups in the nLCEs. In our approach, the parameter *A* physically corresponds to the order parameter that an LCE with no EHA content (i.e., 100% mesogenic content) would be expected to have. Importantly, the fitting was performed using data only for LCEs that did not exhibit phase separation (see images in Figure 7b).

The parameters *A*, c*, and β found from fitting eq 5 to ⟨*P*_2_⟩ and ⟨*P*_4_⟩ against mol % EHA are given in the table, Figure 7c. The materials for which phase separation was observed were nLCE-51 and nLCE-53; these are indicated in Figure 7a with open symbols. Interestingly, the fits to both ⟨*P*_2_⟩ and ⟨*P*_4_⟩ provide a consistent value for the critical concentration, c* of 45 mol % EHA (55 mol % mesogenic content), which is in excellent agreement with the experimental observation that nLCEs fabricated with a mesogenic content below ∼55 mol % exhibit phase separation, Figure 7b. The fitting parameter, *A*, corresponds to ⟨*P*_2_⟩ and ⟨*P*_4_⟩ for LCEs with 100 mol % mesogenic content (0 mol % EHA) and takes values of 0.77 ± 0.01 and 0.42 ± 0.03, respectively.

**Figure 8:**
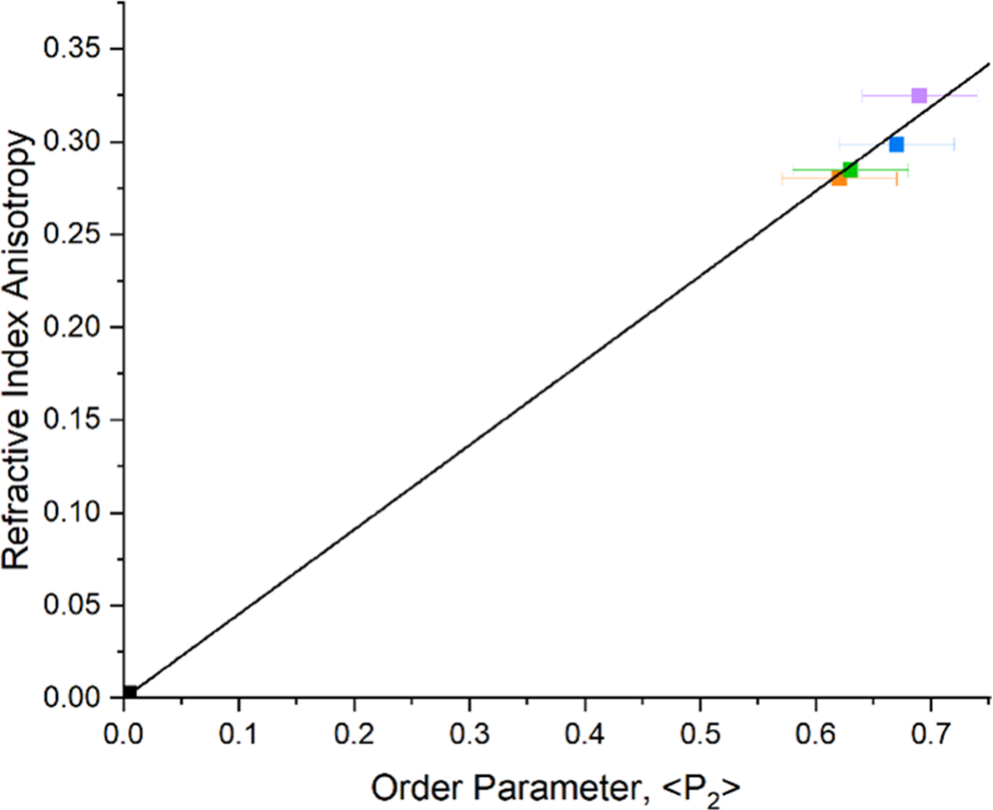
Order parameter and refractive index anisotropy of various mesogenic content nematic LCEs, at room temperature. The linear fit demonstrates the interdependence of these parameters at a fixed temperature, as anticipated by eq 6. The data correspond to nematic LCEs with 72 mol % (purple square), 67 mol % (blue square), 64 mol % (green square), and 62 mol % (orange square) mesogenic content.

We now consider whether the order parameters measured for the different compositions in this family of LCEs can be directly related to their refractive indices. Such a relationship is commonly used to deduce the temperature dependence of the order parameter from refractive indices in low-molar-mass liquid crystals.^24^ Here, a correlation would allow a direct method for controlling the optical anisotropy of the LCEs. The refractive index anisotropy of a liquid crystal,^24^ represented by  in eq 6, relates the refractive indices to the order parameter, ⟨*P*_2_⟩,
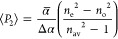
6where Δα = (α_∥_ – α_⊥_) is the difference in polarizability along the extraordinary and ordinary axes, and α̅ = (α_∥_ + 2α_⊥_)/3 is the average polarizability.^24^ The linear interdependence of the refractive index anisotropy, , and the order parameter, ⟨*P*_2_⟩ is shown in Figure 8, for various nLCEs at room temperature.

The applicability of eq 6 to this family of LCEs is both useful and interesting. In general, one would not expect the properties of chemically different liquid crystals to be related in this way, rather a single material will follow such behavior as a function of temperature.^24^ Indeed, Gleeson et al.^24^ showed that materials with very different values of birefringence (ranging from ∼0.05 to ∼0.2) have extremely similar order parameter behavior; it is the temperature dependence of the order parameters and refractive index anisotropy that is correlated. The observation that eq 6 can be used for this family of nLCEs is perhaps to be expected, as eq 5 and Figure 7 demonstrate that concentration plays the role of temperature for this system.

## Conclusions

This work has demonstrated that a family of highly transparent liquid crystal elastomers can be tuned *via* their composition, enabling control of both optical and physical properties. An increased mesogenic content of the LCE precursor mixture resulted in an increased *T*_NI_, and combined with the phase templating at room temperature, this enabled polymerization of nematic LCEs with different nematic order parameters. Indeed, increasing the mesogenic content by 33 mol % caused the order parameters ⟨*P*_2_⟩ and ⟨*P*_4_⟩ to vary from 0.46 to 0.73 and 0.19 to 0.35, respectively. Additionally, the Onsager-type fitting to the data showed a critical concentration of 45 mol % EHA content in this LCE family, setting the upper limit of plasticizer concentration, EHA, for nematic LCE formation.

Through investigations into the optical properties, we have determined factors that may be used for optical tuning of the transparent LCEs. Considering composition first, both *n*_o_ and *n*_e_ of nematic LCEs were shown to increase for an increased mesogenic content of the LCE, with refractive indices changing by up to 0.026 and the birefringence by ∼18% for a 10 mol % increase in the mesogenic content. Temperature studies demonstrated a linear variation of the refractive indices, with temperature coefficients of the refractive index on the order of 10^–4^ K^–1^, close to literature values for other optical plastics. The linear dependence observed allows us to conclude that for such materials, where the LCE *T*_NI_ is sufficiently high (in this case, it was not observed up to 250 °C), the temperature dependence of the refractive indices is dominated by changes in density.

Furthermore, the average refractive index of nematic and isotropic LCEs can be tuned solely via composition (order independent) and can be anticipated based on fittings of the Newton equations to the mole fraction of mesogenic content. Finally, we observe that for nematic LCEs of varied mesogenic content, the relationship between the measured order parameter ⟨*P*_2_⟩ and the refractive index anisotropy can be well predicted. This further demonstrates that the order parameters and optical properties of this acrylate LCE family can be anticipated, which gives the opportunity for precise design. These results give detailed insight into the design of auxetic liquid crystal elastomers for a variety of optical applications, including impact-resistant glass, where it is desirable to be able to design both the average refractive index and the anisotropy, for example, to control optical losses that occur due to Fresnel reflections.

## Data Availability

Data set for this paper can be found at the University of Leeds repository at 10.5518/1399.

## Supporting Information Available

Raman spectroscopy depolarization ratio fitting for a nematic LCE (LCE-56) (Figure S1); transmission spectroscopy of the highly transparent nematic LCE (nLCE-62) (Figure S2); temperature-dependent birefringence of nematic LCEs (Figure S3); and Maier–Saupe fitting to the order parameters of nematic LCEs (Figure S4) (PDF)
