# Supplementary material for: Controlling the Optical Properties of Transparent Auxetic Liquid Crystal Elastomers
Source: Macromolecules. 2024 Feb 19;57(5):2030–8. doi: 10.1021/acs.macromol.3c02226 (PMC10938889; doi:10.1021/acs.macromol.3c02226)
Supplement: Supplementary file 1 — ma3c02226_si_001.pdf [file ma3c02226_si_001.pdf]

Supporting Information for “Controlling the Optical Properties of Transparent Acrylate Liquid Crystal Elastomers”

Emily J. Cooper[1], Matthew Reynolds[1], Thomas Raistrick[1], Stuart R. Berrow[1], Ethan I.L. Jull†[1], Victor Reshetnyak [1,2], Devesh Mistry[1], Helen F. Gleeson*[1]*

[1] School of Physics and Astronomy, University of Leeds, Leeds LS2 9JT, United Kingdom

[2] Taras Shevchenko National University of Kyiv, Kyiv 03680, Ukraine

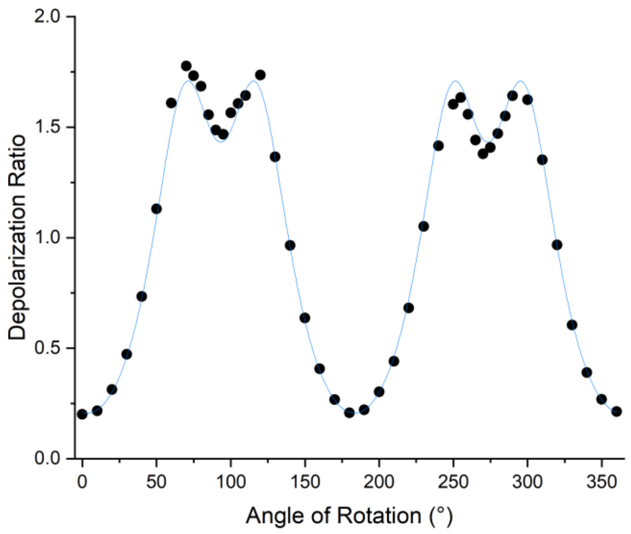

Figure S1. An example of a fitting of *the* depolarization ratio*, calculated from the ratio of* equations *1 and 2,* for nLCE-56 under a full rotation of the sample. Here, the fitting to the depolarization ratio gives parameters of $\langle P_2 \rangle = 0.54 \pm 0.05$, $\langle P_4 \rangle = 0.24 \pm 0.05$ and $r = -0.26 \pm 0.01$.

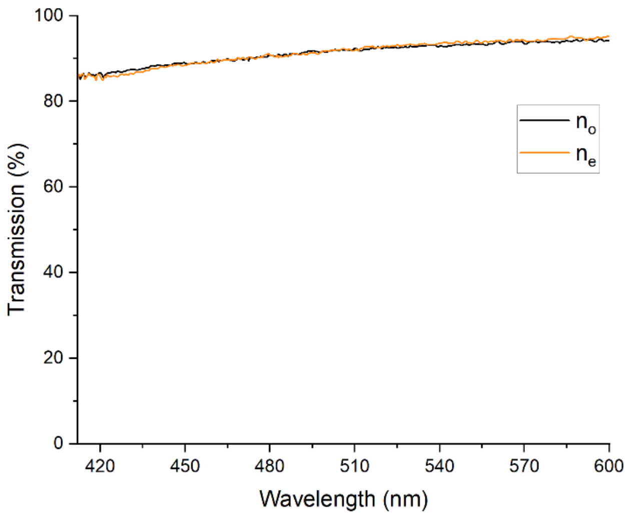

Figure S2. Transmission spectroscopy of a nematic LCE (nLCE-62), measured with the light polarized along both n_o and n_e across visible wavelengths. The data have been corrected for the light losses due to Fresnel reflections. The transmission of the LCE is >94% for both orientations at 589nm.

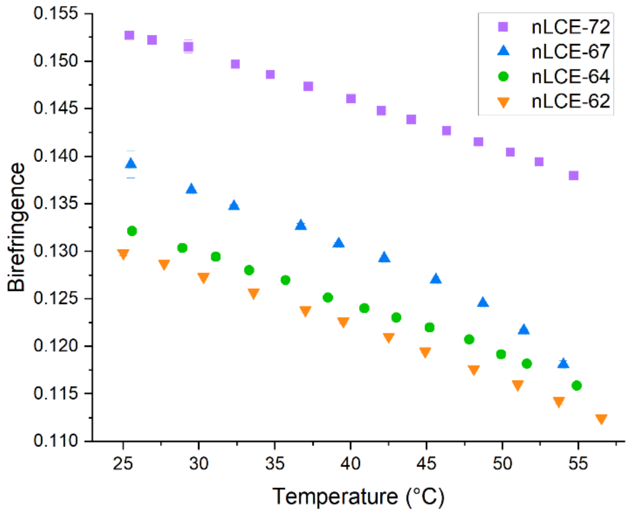

Figure S3. The temperature dependence of the birefringence ($\Delta n = n_e - n_o$) for nematic LCEs with 72 mol. % (purple squares), 67 mol. % (blue triangles), 64 mol. % (green circles) and 62 mol. % (orange triangles) mesogenic content. It can be seen that the birefringence is higher for the LCEs with higher mesogenic content.

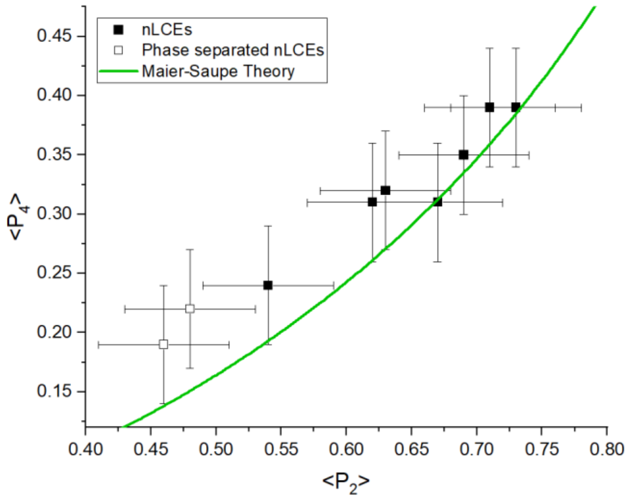

Figure S4. The predicted mean field Maier-Saupe theory[1, 2] is shown in the green curve showing good agreement with the order parameters of nematic LCEs. For reference, nLCE-51 can be seen as the leftmost data point, nLCE-84 is the rightmost data point.

References

(1) Zannoni, C. Order Parameters and Orientational Distributions in Liquid Crystals. In *Polarized Spectroscopy of Ordered Systems*, Samori', B., Thulstrup, E. W. Eds.; Springer Netherlands, 1988; pp 57-83.

(2) Andrienko, D. Introduction to liquid crystals. *J. Mol. Liq.* 2018, *267*, 520-541. DOI: 10.1016/j.molliq.2018.01.175.
